# Circulating neutrophils levels are a predictor of pneumonia risk in chronic obstructive pulmonary disease

**DOI:** 10.1186/s12931-019-1157-0

**Published:** 2019-08-23

**Authors:** Steven J. Pascoe, Alberto Papi, Dawn Midwinter, Sally Lettis, Neil Barnes

**Affiliations:** 1 Global Respiratory Clinical Development, GlaxoSmithKline plc., King of Prussia, USA; 2 Respiratory Medicine, University Hospital St. Anna, Ferrara, Italy; 3 Clinical Statistics, GlaxoSmithKline plc., Uxbridge, UK; 4 Global Respiratory Franchise, GlaxoSmithKline plc., Brentford, UK; 5 William Harvey Institute Barts and the London School of Medicine and Dentistry, London, UK

**Keywords:** COPD pathology, Innate immunity, Neutrophil biology, Pneumonia, Respiratory infection

## Abstract

**Background:**

Patients with chronic obstructive pulmonary disease (COPD) have excess risk of developing pneumonia; however, no definitive biomarkers of risk have been established. We hypothesized that blood neutrophils would help predict pneumonia risk in COPD.

**Methods:**

A meta-analysis of randomized, double-blind clinical trials of COPD patients meeting the following criteria were selected from the GlaxoSmithKline trial registry: ≥1 inhaled corticosteroid-containing (ICS) arm (fluticasone propionate/salmeterol or fluticasone furoate/vilanterol), a control arm (non-ICS), pre-randomization blood neutrophil counts, ≥24-week duration. The number of patients with pneumonia events and time to first event (Kaplan–Meier analysis) were evaluated (post-hoc), stratified by baseline blood neutrophil count and ICS use. A Cox proportional hazards model was used to calculate hazard ratios (HR), split by median baseline blood neutrophils.

**Results:**

Ten studies (1998 to 2011) with 11,131 patients were identified. The ICS (*n* = 6735) and non-ICS (*n* = 4396) cohorts were well matched in neutrophil distributions and demographics. Increasing neutrophil count was associated with an increased proportion of patients with pneumonia events; patients below the median neutrophil count were at less risk of a pneumonia event (HR, 0.75 [95% confidence interval 0.61–0.92]), and had longer time to a first event, compared with those at/above the median. The increase in pneumonia risk by neutrophil count was similar between the two cohorts.

**Conclusions:**

Increased blood neutrophils in COPD were associated with increased pneumonia risk, independent of ICS use. These data suggest blood neutrophils may be a useful marker in defining treatment pathways in COPD.

**Electronic supplementary material:**

The online version of this article (10.1186/s12931-019-1157-0) contains supplementary material, which is available to authorized users.

## Background

Patients with chronic obstructive pulmonary disease (COPD) are at a heightened risk of pneumonia compared with otherwise healthy individuals and often have worse clinical outcomes in terms of pneumonia severity [1]. However, the effect of COPD on pneumonia-related mortality remains unclear [2–4]. Inhaled corticosteroid (ICS) therapy is effective at reducing exacerbations in patients with COPD [5], particularly when combined with a long-acting β_2_ agonist (LABA) [6, 7]. However, ICS-containing treatments have been associated with an increased risk of pneumonia in patients with COPD [6–9]. The level of exacerbation reduction afforded by ICS is influenced by eosinophil count, a marker of inflammatory response. In a post-hoc analysis, patients with ≥2% blood eosinophils or ≥150 eosinophils/mm^3^ were found to have a significant reduction in the rate of moderate/severe exacerbations upon the addition of ICS to LABA therapy, which was not observed in those with <2% blood eosinophils or <150 eosinophils/mm^3^ [10]. Blood eosinophil count has also been evaluated for its association with pneumonia risk and, as such, offers potential as a biomarker to assess COPD-related pneumonia events; however, findings have been mixed [10–13]. Circulating blood neutrophils have been observed to be present in higher concentrations in patients with stable COPD [14] and, like eosinophils, their level can increase during COPD exacerbations [6, 15]. There is robust evidence for the participation of neutrophils in the immune response to pneumonia, with both potential beneficial and detrimental effects [16]. Although neutrophils have been identified as an effector cell in the pathogenesis of COPD, little is known as to how much of the increase in circulating neutrophils in COPD is driven by the underlying inflammatory process, as opposed to a response to bacterial colonization and/or infection in the airways. We hypothesized that blood neutrophil levels could be reflective of changes in the microbiome that are associated with the risk of developing pneumonia. Therefore, the demonstration of an association between blood neutrophil levels and pneumonia risk has potential to both inform on the biological basis for the increased risk of developing pneumonia in COPD and to provide a prospective biomarker for risk stratification.

In this post-hoc study, we examined patient data across 10 clinical trials to evaluate the association between circulating blood neutrophils and pneumonia risk in patients with COPD.

## Methods

### Study objectives

This post-hoc meta-analysis evaluated data derived from 10 clinical trials within the GlaxoSmithKline plc. (GSK) clinical trial registry to assess the potential relationship between baseline blood neutrophil count and incidence of pneumonia in patients with moderate or severe COPD.

### Search criteria and studies

The GSK clinical trials database was searched for GSK-sponsored trials of ≥24 weeks’ duration with evaluable, patient-level data for baseline neutrophil and eosinophil counts. Selected trials were randomized, double-blind, clinical trials of COPD patients with at least one ICS-containing arm, a control arm (non-ICS containing), and pre-randomization measurements of blood neutrophil and eosinophil counts. Search criteria specified that a trial must include a fluticasone furoate (FF) plus vilanterol (VI) or fluticasone propionate (FP) plus salmeterol (SAL) arm. Therefore, the ICS-containing treatments considered for this study were FF or FP alone or in combination with a LABA, and the non-ICS-containing treatments included any treatment not containing FF or FP.

Patients included in these studies were aged ≥40 years, had an established diagnosis of COPD, and a smoking history of ≥10 pack-years, in line with American Thoracic Society/European Respiratory Society guidelines [17].

### Analyses

Clinically-diagnosed pneumonia adverse events, which were re-coded according to the Medical Dictionary for Regulatory Activities (MedDRA version 15.1), were identified in patient-level data; Additional file 1: Table S1 contains a list of the terms used. The relationship between baseline neutrophil count and pneumonia risk was assessed using three post-hoc comparisons. Neutrophil subgroups (<median vs. ≥median) were compared with treatment as a covariate (ICS-containing vs. non-ICS-containing) to assess the relationship averaged across all patients. The analysis was repeated for ICS-treated patients only and for non-ICS-treated patients only. Additionally, pneumonia risk was evaluated by neutrophil:eosinophil ratio (<median vs. ≥median) and neutrophil:leukocyte ratio (<median vs. ≥median).

For these analyses, a Cox proportional hazards model was used to calculate hazard ratios (HR), stratifying by trial with a term for baseline blood neutrophil subgroup. Heterogeneity across trials was tested for each of the analyses by fitting an additional model, which included a term for neutrophil subgroup by trial interaction. Within each trial treatment by neutrophil subgroup, interaction was also assessed. SAS versions 9.4 or earlier were used for the analyses. Kaplan–Meier plots were used to evaluate time to first pneumonia event by baseline neutrophil count (quartiles and by <median or ≥median) based on a cut-off used in a previous study) [10].

## Results

### Studies

A total of 10 studies conducted between 1998 and 2011, containing 11,131 patients, were identified (NCT01009463, NCT01017952, NCT01053988, NCT00361959, SFCB3024, NCT01054885, SCO100470, SCO30002, SFCA3006, SFCA3007). A summary of these studies is provided in Table 1. All studies used fixed dose(s) of ICS.

**Table 1:** Summary of clinical trials included in these analyses

	ITT patients (*N)*	Trial duration (weeks)	Treatment arms	Primary endpoint
Dransfield et al. HZC102871 (NCT01009463) [18]	1622	52	FF/VI: 50/25 μg, 100/25 μg, 200/25 μg (QD) VI: 25 μg (QD)	Annual rate of moderate and severe COPD exacerbations
Dransfield et al. HZC102970 (NCT01017952) [18]	1633	52	FF/VI: 50/25 μg, 100/25 μg, 200/25 μg (QD) VI: 25 μg (QD)	Annual rate of moderate and severe COPD exacerbations
Kerwin et al. HZC112206 (NCT01053988) [19]	1030	24	FF/VI: 50/25 μg, 100/25 μg (QD) FF: 100 μg (QD) VI: 25 μg (QD)	Change from baseline in weighted mean FEV_1_ over 0–4 h post-dose at Day 168; Change from baseline in clinic visit trough (pre-bronchodilator and predose) FEV_1_ at Day 169
INSPIRE SCO40036 (NCT00361959) [9]	1323	104	FP/SAL: 500/50 μg (BD) TIO: 18 μg (QD)	Rate of healthcare-utilization-based exacerbations of COPD
TRISTAN SFCB3024 [20, 21]	1465	52	FP/SAL: 500/50 μg (BD) FP: 500 μg (BD) SAL: 50 μg (BD)	Pre-bronchodilator FEV_1_ at Week 52
Martinez et al. HZC112207 (NCT01054885) [22]	1224	24	FF/VI: 100/25 μg, 200/25 μg (QD) FF: 100 μg, 200 μg (QD) VI: 25 μg (QD)	Change from baseline in weighted mean FEV_1_ over 0–4 h post-dose at Day 168; change from baseline in trough FEV_1_ at Day 169
SCO100470 [23]	1050	24	FP/SAL: 250/50 μg (BD) SAL: 50 μg (BD)	Mean trough FEV_1_ at endpoint*, mean TDI focal score at endpoint*
SCO30002 [24]	387	52	FP/SAL: 500/50 μg (BD) FP: 500 μg (BD)	Time from the start of treatment to the first moderate or severe exacerbation
Mahler et al. SFCA3006 [25]	674	24	FP/SAL: 500/50 μg (BD) FP: 500 μg (BD) SAL: 50 μg (BD)	Mean change from baseline in AM pre-dose and 2-h post-dose FEV_1_
Hanania et al. SFCA3007 [26, 27]	723	24	FP/SAL: 250/50 μg (BD) FP: 250/50 μg (BD) SAL: 50 μg (BD)	Mean change from baseline in AM pre-dose and 2-h post-dose FEV_1_

*Definition of abbreviations: AM* morning; *BD* twice daily; *BDI* Baseline Dyspnoea Index; *CBSQ* Chronic Bronchitis Symptoms Questionnaire; *CRDQ* Chronic Respiratory Disease Questionnaire; *CRQ-SAS* Chronic Respiratory Questionnaire Self-Administered Standardized; *FEV*_*1*_ forced expiratory volume in 1 s; *FF* fluticasone furoate; *FP* fluticasone propionate; *FVC* forced vital capacity; *ITT* intent-to-treat; *QD* once daily; *SAL* salmeterol; *SGRQ* St George’s Respiratory Questionnaire; *TDI* Transition Dyspnoea Index

*Last available on-treatment value

Evaluable baseline neutrophil data were available for 10,842 patients. Further information for each study is available from the corresponding primary publications, or the GSK clinical trial registry [9, 18–27]. All patients provided written informed consent and the included studies were approved by the appropriate institutional review boards at all study sites. Of the 10 studies examined, four assessed the use of FF/VI and six the use of FP/SAL.

### Patients

**Fig. 1:**
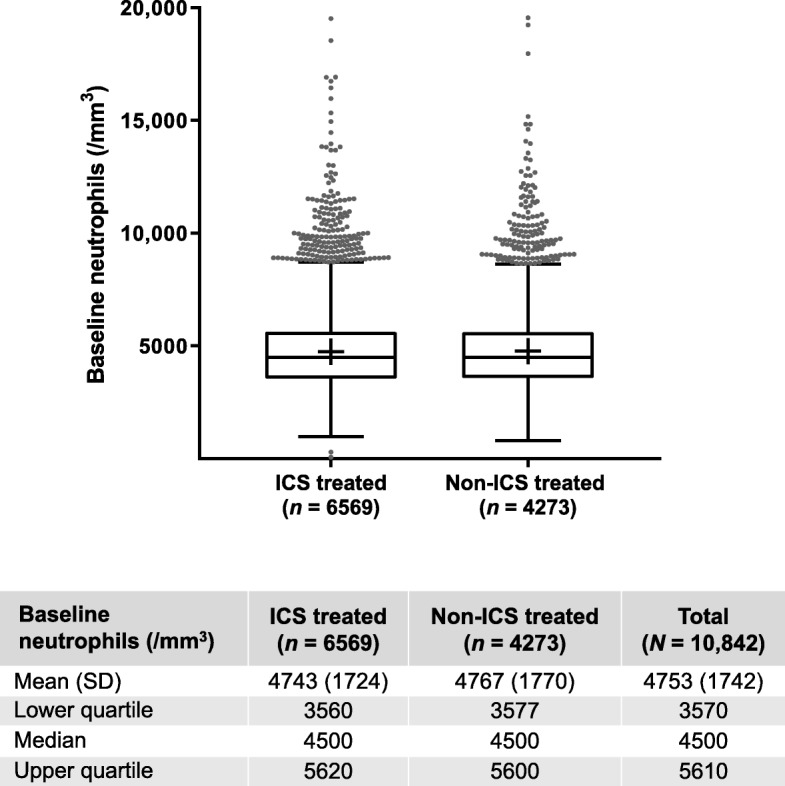
Distribution of baseline neutrophils across all studies by ICS or non-ICS-containing treatment. The “+” marks show the mean values. The grey dots represent outliers. Definition of abbreviations: *ICS* inhaled corticosteroid; *SD* standard deviation

Across all studies, treatment groups, and neutrophil quartiles, patients were well matched in terms of demographic characteristics such as age and body mass index (BMI); there was a slightly higher proportion of male versus female patients (Additional file 1: Table S2). Mean, median, lower quartile, and upper quartile values for baseline neutrophils are shown in Fig. 1; neutrophil distributions were similar between patients in the ICS-containing and non-ICS-containing groups.

No differences in the severity of COPD (as measured by percentage predicted forced expiratory volume in 1 s [FEV_1_]) were observed between the high and low neutrophil groups. Furthermore, the median neutrophil count at study entry was similar across the studies, ranging from 4208.39–4950.00 in total (Additional file 1: Table S3) and did not appear to be higher in studies that recruited patients with COPD exacerbation history (HZC102871, HZC102970, SFCB3024, SCO40036).

A total of 46% of the study population were current smokers. There was no major difference in the distribution of neutrophils between current and former smokers; current smokers had a slightly higher mean/median than former smokers (4700/mm^3^ versus 4300/mm^3^, Additional file 1: Figure S1), however current smokers and former smokers were similarly represented in the high versus low neutrophil groups.

### Neutrophil count and pneumonia risk

**Fig. 2:**
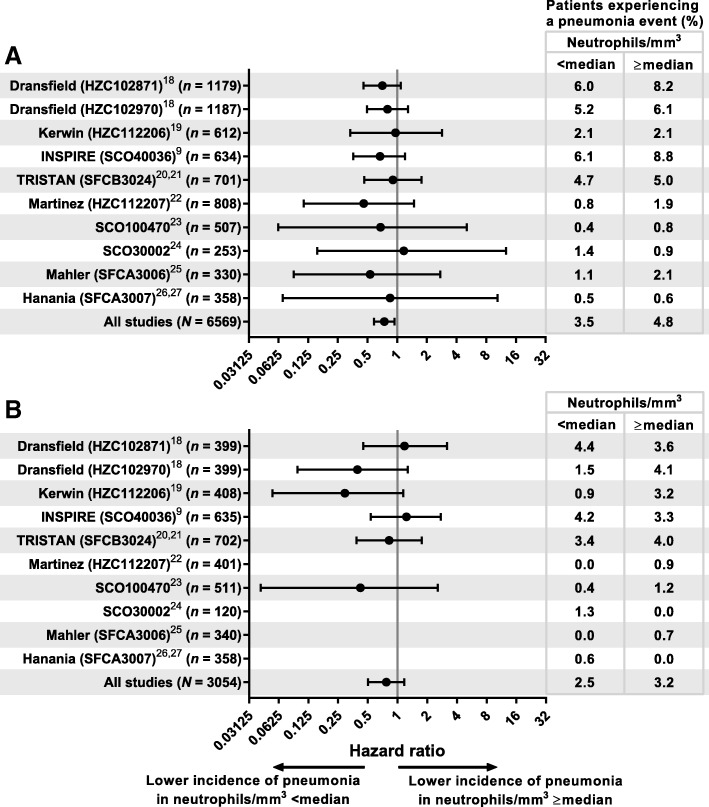
Effect of median neutrophil subgroup on pneumonia events. Definition of abbreviation: *ICS* inhaled corticosteroid. Note: (**a**) ICS-treated groups and (**b**) non-ICS-treated groups. Analyzed using a Cox Proportional Hazards model, stratified by study with term for neutrophil group. Studies with 0% incidence in either subgroup were not formally analyzed and were not included in the all studies analysis. Error bars represent the 95% CI

In general, pneumonia incidence was lower in patients treated with non-ICS-containing therapies versus those on ICS (Table 2 and Fig. 2), and in patients with baseline neutrophil counts below the median. Stratifying by presence of ICS showed that patients with lower neutrophil counts had similar reductions in pneumonia risk versus those with higher neutrophil counts irrespective of whether their treatment was ICS- or non-ICS-containing (ICS-containing: HR, 0.74 [0.58–0.94, *P* = 0.015]; non-ICS-containing: HR, 0.77 [0.50–1.17], *P* = 0.228) (Fig. 2). In keeping with this observation, the interaction test between neutrophils and ICS treatment was non-significant for each individual study and for the total cohort of this analysis *(P* = 0.80). The numbers of patients experiencing ≥1 pneumonia adverse event are provided in Table 2, grouped by baseline neutrophil count and treatment, and presented by pneumonia severity.

**Table 2:** Number of patients with ≥1 pneumonia adverse event in the treatment arms of the 10 included studies, by blood neutrophil count and treatment subgroups

Trials (duration)	Blood neutrophil count: <median, *n* (%)				Blood neutrophil count: ≥median, *n* (%)			
	*n*	Pneumonia	Serious pneumonia	Fatal pneumonia	*n*	Pneumonia	Serious pneumonia	Fatal pneumonia
NCT01009463^+Exac^								
(52 weeks) [18]								
FF/VI 50/25	178	12 (6.7)	6 (3.4)	0	214	17 (7.9)	8 (3.7)	0
FF/VI 100/25	210	13 (6.2)	8 (3.8)	0	186	12 (6.5)	3 (1.6)	0
FF/VI 200/25	182	9 (4.9)	2 (1.1)	2 (1.1)	209	21 (10.0)	11 (5.3)	4 (1.9)
VI 25	203	9 (4.4)	2 (1.0)	0	196	7 (3.6)	0	0
NCT01017952^+Exac^								
(52 weeks) [18]								
FF/VI 50/25	210	8 (3.8)	5 (2.4)	0	190	10 (5.3)	4 (2.1)	0
FF/VI 100/25	189	11 (5.8)	5 (2.6)	0	200	14 (7.0)	8 (4.0)	1 (0.5)
FF/VI 200/25	214	13 (6.1)	5 (2.3)	0	184	11 (6.0)	4 (2.2)	0
VI 25	206	3 (1.5)	2 (1.0)	0	193	8 (4.1)	4 (2.1)	1 (0.5)
NCT01053988^-Exac^								
(24 weeks) [19]								
PBO	113	1 (0.9)	0	0	94	2 (2.1)	1 (1.1)	0
FF/VI 50/25	108	3 (2.8)	1 (0.9)	0	96	0	0	0
FF/VI 100/25	109	2 (1.8)	1 (0.9)	0	96	4 (4.2)	0	0
VI 25	107	1 (0.9)	1 (0.9)	0	94	4 (4.3)	2 (2.1)	0
FF 100	110	2 (1.8)	2 (1.8)	0	93	2 (2.2)	1 (1.1)	0
NCT01054885^-Exac^								
(24 weeks) [22]								
PBO	96	0	0	0	109	0	0	0
FF/VI 100/25	103	0	0	0	100	1 (1.0)	0	0
FF/VI 200/25	82	0	0	0	120	4 (3.3)	3 (2.5)	0
VI 25	92	0	0	0	104	2 (1.9)	2 (1.9)	0
FF 100	94	1 (1.1)	0	0	108	2 (1.9)	0	0
FF 200	106	2 (1.9)	1 (0.9)	0	95	1 (1.1)	1 (1.1)	0
NCT00361959^-Exac/+Exac^								
(104 weeks) [9]								
FP/SAL 500/50	246	15 (6.1)	11 (4.5)	1 (0.4)	388	34 (8.8)	30 (7.7)	2 (0.5)
TIO	236	10 (4.2)	8 (3.4)	0	399	13 (3.3)	11 (2.8)	0
SFCB3024^+Exac^								
(52 weeks) [20, 21]								
PBO	181	3 (1.7)	1 (0.6)	0	166	5 (3.0)	2 (1.2)	0
FP/SAL 500/50	187	9 (4.8)	4 (2.1)	0	154	7 (4.5)	3 (1.9)	0
SAL 50	197	10 (5.1)	6 (3.0)	0	158	8 (5.1)	4 (2.5)	0
FP 500	176	8 (4.5)	4 (2.3)	0	184	10 (5.4)	5 (2.7)	0
SCO100470^-Exac^								
(24 weeks) [23]								
FP/SAL 250/50	241	1 (0.4)	0	0	266	2 (0.8)	2 (0.8)	0
SAL 50	256	1 (0.4)	1 (0.4)	0	255	3 (1.2)	3 (1.2)	0
SCO30002^-Exac/+Exac^								
(52 weeks) [24]								
PBO	78	1 (1.3)	1 (1.3)	0	42	0	0	0
FP/SAL 500/50	78	2 (2.6)	0	0	51	1 (2.0)	0	0
FP 500	67	0	0	0	57	0	0	0
SFCA3006^-Exac^								
(24 weeks) [25]								
PBO	104	0	0	1 (1.0)	76	1 (1.3)	0	0
FP/SAL 500/50	84	1 (1.2)	1 (1.2)	0	79	1 (1.3)	1 (1.3)	0
SAL 50	91	0	0	0	69	0	0	0
FP 500	104	1 (1.0)	1 (1.0)	0	63	2 (3.2)	1 (1.6)	0
SFCA3007^-Exac^								
(24 weeks) [26, 27]								
PBO	89	0	0	0	92	0	0	0
FP/SAL 250/50	102	0	0	0	74	0	0	0
SAL 50	86	1 (1.2)	1 (1.2)	0	91	0	0	0
FP 250	90	1 (1.1)	0	0	92	1 (1.1)	1 (1.1)	0

*Definition of abbreviations: FF* fluticasone furoate; *VI* vilanterol; *PBO* placebo; *SAL* salmeterol; *TIO* tiotropium.^**-**Exac^study conducted in patients without a history of COPD exacerbations; ^+Exac^study conducted in patients with a history of exacerbations. Pneumonia/serious pneumonia includes on-treatment events as defined in each individual study. Fatal pneumonia includes on- and post-treatment events

**Fig. 3:**
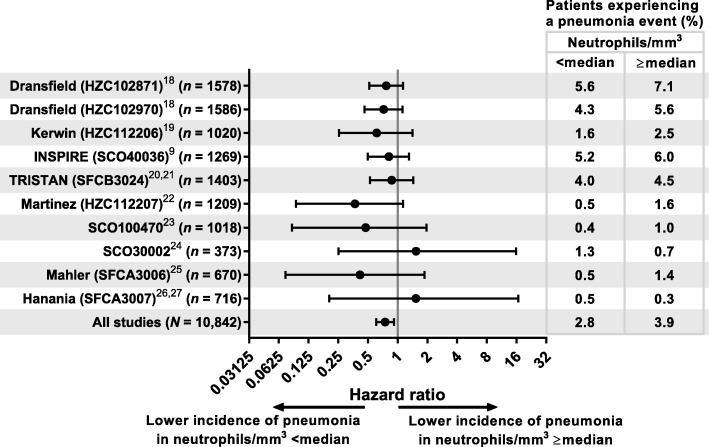
Effect of median neutrophil subgroup on pneumonia events. Note: Analyzed using a Cox proportional hazards model, stratified by study with terms for treatment and neutrophil group. Treatments were defined as ICS containing or non-ICS containing. Error bars represent the 95% CI

Across all studies, a lower proportion of patients below the median neutrophil count had pneumonia events compared with those at or above the median (2.8% vs. 3.9%; HR, 0.75, [95% CI 0.61–0.92]) (Fig. 3). This pattern was observed in eight of the 10 trials analyzed, with the exceptions occurring in two of the trials with smaller populations (SCO30002 [*n* = 373], SFCA3007 [*n* = 716]) (Fig. 3). The test of neutrophils by trial interaction (heterogeneity) was not significant *(P* = 0.94), so there was no statistical evidence of an inconsistent effect of neutrophil levels on pneumonia incidence across the trials. Exacerbation histories for each study are included in Additional file 1: Table S2. The four studies with the highest incidence of pneumonia (HZC102871, HZC102970, SFCB3024 and SCO40036) had, as an entry requirement, a history of exacerbations. The six studies with the lowest incidence of pneumonia (HZC112206, HZC112207, SFCA3006, SFCA3007, SCO30002 and SCO100470) did not require patients to have an exacerbation history.

**Fig. 4:**
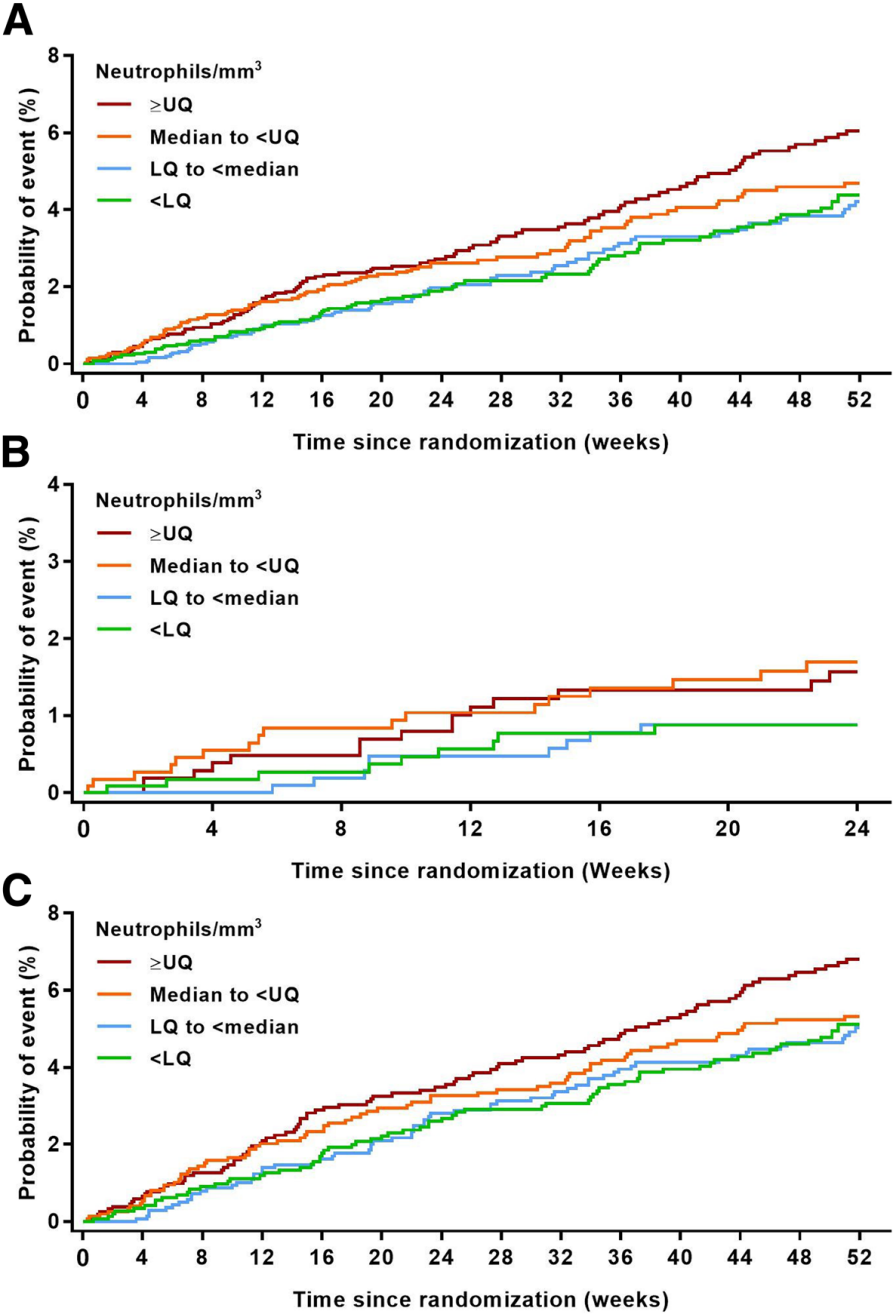
Kaplan–Meier plot of time to first pneumonia event by absolute neutrophil count. Definition of abbreviations: *LQ* lower quartile; *UQ* upper quartileNote: (**a**) All studies, (**b**) 24-week studies only and (**c**) 52-week data from 52-week and 104-week studies only

The Kaplan–Meier plots up to Week 52 showed that patients in the highest baseline neutrophil quartile had an increased risk of pneumonia compared with the lower three quartiles, which all showed similar levels of pneumonia risk at Week 52 (Fig. 4). Kaplan–Meier analysis of the 24-week studies showed that pneumonia risk was higher at Week 24 in patients with baseline neutrophil counts at or above the median versus those below the median (Fig. 4).

## Discussion

Across eight of the 10 clinical studies included within this analysis, increased circulating neutrophils in patients with stable COPD were associated with an increased risk of pneumonia. A greater incidence of pneumonia was observed in patients with baseline blood neutrophil counts at or above the median value when compared with those below the median. As has been shown previously [28], the ICS-containing therapies conveyed an increased risk of pneumonia versus those without ICS. Notably, however, the difference in pneumonia risk between patients at or above the median neutrophil count versus those below the median remained consistent regardless of the presence of ICS. Furthermore, the interaction test for the effects of ICS and neutrophil counts was found to be non-significant. Taken together, these results suggest that blood neutrophils could potentially be a useful biomarker for informing physicians if a patient has a high risk of pneumonia, when considering ICS therapy. It should be noted that this is the first study to investigate the use of neutrophil count as a potential biomarker for pneumonia risk in patients with COPD.

In addition to neutrophils, studies have demonstrated that eosinophils are biomarkers that also have potential to assess COPD-related pneumonia events [10–12], but the association between eosinophils and pneumonia risk may not be as clear as that between neutrophils and pneumonia risk. With this in mind, it is possible that blood neutrophil count may be a more reliable indicator of pneumonia risk in patients with COPD than blood eosinophil count, but further research would be of benefit in support of this claim.

This study provides evidence of a clear association between raised neutrophil counts and increased pneumonia risk but does not provide any information on the reason behind the association. In line with other meta-analyses in this area of research [28], where associations are identified and hypotheses generated, further investigations in appropriately designed studies to evaluate the potential role of other variables, including the role of concomitant comorbidities, are crucially needed. It is interesting to note that patients with neutrophil counts above and below the median had strikingly similar demographics and, importantly, exacerbation and hospitalization rates prior to study entry. A possible link between pneumonia and neutrophil counts could be the presence of potentially pathogenic airway bacteria driving both a neutrophilic response and an increased risk of pneumonia. However, the lack of a relationship between neutrophil levels and exacerbation rates is somewhat discordant with this hypothesis, and only further studies of the airway microbiome, infection risk and neutrophil counts are likely to elucidate the nature of this relationship.

A major strength of this study was that the analyses were performed using a large patient population with evaluable baseline neutrophil data. Additionally, the definition of pneumonia that was applied within the 10 included studies was uniform and used predefined MedDRA terms, though a limitation may be that these relatively broad terms could have mislabeled comorbidities as pneumonia events. Another limitation was that only GSK studies with patient-level data were included in the analysis. By limiting the evaluated studies to those within the GSK clinical trials registry that included an ICS-containing arm, only COPD therapies containing FF and FP were assessed; future investigations should explore the relationship between neutrophils and pneumonia risk across a range of ICS and non-ICS-containing COPD therapies. It is worth noting that the INSPIRE study was unique within the current analysis for its inclusion of a potentially active tiotropium comparator arm, which could have impacted on patients’ risk of pneumonia, or the relationship between neutrophils and pneumonia risk [9]; however, the strength and direction of the relationship between neutrophils and pneumonia risk in INSPIRE was consistent with that observed in the majority of the evaluated studies.

This study was post-hoc, and was not powered for statistical significance. As patients not on ICS had fewer pneumonia events, the non-ICS group may have been insufficiently powered to show a clear relationship between baseline neutrophils and pneumonia risk. Likewise, mortality was low in the evaluated studies, preventing any analyses on the relationship between neutrophils and pneumonia-related deaths.

When interpreting the findings from this study, the degree of heterogeneity that exists between the trials included in these analyses in terms of duration, treatment and patient populations should be considered, as should their exclusion of patients with mild COPD.

A limitation of this study was that, blood neutrophil count was measured at a single time point during screening. The lack of multiple neutrophil count measurements precludes evaluation of both the reproducibility of blood neutrophil measurements in patients with COPD, and the effect of change in neutrophil count on pneumonia risk.

Additionally, exposure to ICS at baseline was not analyzed as part of the studies that form this analysis; however, given that all 10 trials were randomized studies, it is unlikely that ICS exposure at baseline would have affected outcomes.

Studies of pneumonia risk in COPD have demonstrated that BMI and previous history of pneumonia are important factors in determining pneumonia risk [29, 30]; however, pneumonia events prior to screening were not recorded for these trials, and our analyses did not evaluate the effect of variation in BMI on the relationship between neutrophil count and pneumonia risk. These interactions now need to be investigated, as do the relationships between perturbations in the airway microbiome in COPD and pneumonia risk, and between the airway microbiome and blood neutrophil count. Future investigations into the association between neutrophil count and pneumonia risk in patients with moderate/severe COPD would benefit from multiple blood neutrophil measurements over time, prespecified pneumonia endpoints, and standardized, radiological verification of pneumonia.

## Conclusions

Increased circulating blood neutrophils in COPD were associated with increased subsequent risk of pneumonia independent of ICS use. These data suggest that blood neutrophil count may be a useful novel marker to help define treatment pathways in moderate/severe COPD; additional work is needed to further assess this relationship.

## Additional file

Additional file 1: **Table S1.** Preferred terms for pneumonia adverse events. **Table S2.** Patient characteristics at screening by blood neutrophil count, and treatment subgroups. **Table S3.** Summary of median neutrophil counts (/mm^3^) by ICS use and study. **Figure S1.** Boxplot of baseline neutrophils (mm^3^) by smoking status at screening. (DOCX 89 kb)

## Data Availability

Anonymized individual participant data from the studies listed within this publication and their associated documents can be requested for further research from (www.clinicalstudydatarequest.com).

## Abbreviations

AM: morning
BD: twice daily
BDI: Baseline Dyspnoea Index
BMI: body mass index
CBSQ: Chronic Bronchitis Symptoms Questionnaire
COPD: chronic obstructive pulmonary disease
CRDQ: Chronic Respiratory Disease Questionnaire
CRQ-SAS: Chronic Respiratory Questionnaire Self-Administered Standardized
FEV_1_: Forced expiratory volume in 1 s
FF: Fluticasone furoate
FP: Fluticasone propionate
FVC: Forced vital capacity
GSK: GlaxoSmithKline plc.
HR: Hazard ratio
ICS: Inhaled corticosteroid
ITT: Intent-to-treat
LABA: Long-acting β_2_ agonist
PBO: Placebo
QD: Once daily
SAL: Salmeterol
SGRQ: St George’s Respiratory Questionnaire
TDI: Transition Dyspnoea Index
TIO: Tiotropium
VI: Vilanterol
