# Supplementary material for: Circulating neutrophils levels are a predictor of pneumonia risk in chronic obstructive pulmonary disease
Source: Respir Res. 2019 Aug 23;20:195. doi: 10.1186/s12931-019-1157-0 (PMC6708190; doi:10.1186/s12931-019-1157-0)

**Pascoe S, et al. Circulating neutrophils levels are a predictor of pneumonia risk in chronic obstructive pulmonary disease**

**Supplementary Table S1** Preferred terms for pneumonia adverse events

| **MedDRA preferred term** | | | |
| --- | --- | --- | --- |
| Acute pulmonary histoplasmosis | Lung consolidation | Pneumonia chlamydial | Pneumonia parainfluenzae viral |
| Atypical mycobacterial pneumonia | Lung infection | Pneumonia cryptococcal | Pneumonia salmonella |
| Atypical pneumonia | Lung infection pseudomonal | Pneumonia cytomegaloviral | Pneumonia staphylococcal |
| Blastomycosis | Miliary pneumonia | Pneumonia escherichia | Pneumonia streptococcal |
| Bronchopneumonia | Mycobacterium test positive | Pneumonia fungal | Pneumonia toxoplasmal |
| Bronchopneumopathy | Nocardiosis | Pneumonia haemophilus | Pneumonia tularaemia |
| Candida pneumonia | Organizing pneumonia | Pneumonia helminthic | Pneumonia viral |
| Coccidioidomycosis | Pneumocystis jiroveci pneumonia | Pneumonia herpes viral | Pneumonic plague |
| Cryptococcosis | Pneumonia | Pneumonia influenzal | Pneumonitis |
| Empyema | Pneumonia adenoviral | Pneumonia klebsiella | Pulmonary tuberculosis |
| Enterobacter pneumonia | Pneumonia anthrax | Pneumonia legionella | Pyopneumothorax |
| Histoplasmosis | Pneumonia aspiration | Pneumonia measles | Q-fever |
| Infectious pleural effusion | Pneumonia bacterial | Pneumonia moraxella | Tuberculosis |
| Legionella test positive | Pneumonia blastomyces | Pneumonia mycoplasmal |  |
| Lobar pneumonia | Pneumonia bordetella | Pneumonia necrotizing |  |

**Supplementary Table S2** Patient characteristics at screening by blood neutrophil count, and treatment subgroups

|  | **Placebo** | **FF/VI 50/25** | **FF/VI 100/25** | **FF/VI 200/25** | **VI**  **25** | **FF**  **100** | **FF**  **200** | **FP/SAL**  **250/50** | **FP/SAL**  **500/50** | **SAL**  **50** | **FP**  **250** | **FP**  **500** | **TIO**  **18** | **Total** |
| --- | --- | --- | --- | --- | --- | --- | --- | --- | --- | --- | --- | --- | --- | --- |
| N | 1,264 | 1,026 | 1,216 | 1,016 | 1,226 | 410 | 203 | 696 | 1,312 | 1,241 | 183 | 673 | 665 | 11,131 |
| N with available neutrophil count | 1,240 | 996 | 1,193 | 991 | 1,195 | 405 | 201 | 683 | 1,267 | 1,203 | 182 | 651 | 635 | 10,842 |
| Blood neutrophil count: <lower quartile | | | | | | | | | | | | | | |
| n | 328 | 260 | 299 | 234 | 309 | 108 | 59 | 152 | 304 | 318 | 53 | 178 | 103 | 2,705 |
| Mean age, years [SD] | 64.0 [8.81] | 63.3 [9.56] | 63.5 [8.85] | 63.7 [9.61] | 63.8 [9.15] | 65.2 [8.93] | 61.4 [8.23] | 64.3 [9.24] | 63.5 [8.80] | 63.4 [9.46] | 64.0 [8.10] | 64.2 [8.90] | 63.7 [8.81] | 63.7 [9.08] |
| Gender, male, n (%) | 240 (73) | 150 (58) | 159 (53) | 122 (52) | 186 (60) | 67 (62) | 42 (71) | 115 (75) | 236 (78) | 228 (72) | 35 (66) | 118 (66) | 78 (76) | 1,776 (66) |
| Mean BMI, kg/m^2^ | 26.07 | 26.41 | 26.45 | 25.62 | 25.61 | 25.41 | 26.22 | 25.74 | 25.61 | 26.15 | 27.70 | 25.88 | 25.55 | 25.97 |
| Exacerbations requiring antibiotics and/or OCS, within prior year,^*^ total n  n (%): 0  1  2  >2 | 131  96 (73)  20 (15)  11 (8)  4 (3) | 260  58 (22)  130 (50)  55 (21)  17 (7) | 299  97 (32)  145 (48)  35 (12)  22 (7) | 234  43 (18)  139 (59)  39 (17)  13 (6) | 309  106 (34)  137 (44)  43 (14)  23 (7) | 108  80 (74)  25 (23)  2 (2)  1 (<1) | 59  41 (69)  14 (24)  1 (2)  3 (5) | 104  70 (67)  16 (15)  9 (9)  9 (9) | 152  54 (36)  42 (28)  31 (20)  25 (16) | 126  83 (66)  24 (19)  14 (11)  5 (4) | NM  NM  NM  NM  NM | 39  20 (51)  8 (21)  6 (15)  5 (13) | 103  38 (37)  38 (37)  13 (13)  14 (14) | 1,924  786 (41)  738 (38)  259 (13)  141 (7) |
| Exacerbations requiring hospitalization, within prior year,^*^ total n  n (%): 0  1  2  >2 | 131  122 (93)  9 (7)  0  0 | 260  210 (81)  38 (15)  10 (4)  2 (<1) | 299  240 (80)  46 (15)  9 (3)  4 (1) | 234  191 (82)  38 (16)  4 (2)  1 (<1) | 309  266 (86)  38 (12)  3 (<1)  2 (<1) | 108  97 (90)  10 (9)  1 (<1)  0 | 59  52 (88)  7 (12)  0  0 | 104  93 (89)  10 (10)  1 (<1)  0 | 152  116 (76)  32 (21)  3 (2)  1 (<1) | 126  117 (93)  8 (6)  1 (<1)  0 | NM  NM  NM  NM  NM | 39  35 (90)  4 (10)  0  0 | 103  84 (82)  14 (14)  4 (4)  1 (<1) | 1,924  1,623 (84)  254 (13)  36 (2)  11 (<1) |
| Blood neutrophil count: ≥lower quartile–<median | | | | | | | | | | | | | | |
| n | 333 | 236 | 312 | 244 | 299 | 96 | 47 | 189 | 291 | 312 | 37 | 169 | 133 | 2,698 |
| Mean age, years [SD] | 63.9 [8.67] | 63.9 [9.26] | 64.1 [8.99] | 63.4 [8.84] | 63.5 [9.65] | 62.3 [8.54] | 62.6 [9.56] | 63.1 [9.32] | 63.4 [9.12] | 64.1 [8.77] | 65.2 [9.52] | 64.0 [8.70] | 64.1 [8.39] | 63.7 [9.00] |
| Gender, male, n (%) | 242 (73) | 150 (64) | 210 (67) | 138 (57) | 182 (61) | 68 (71) | 37 (79) | 136 (72) | 226 (78) | 216 (69) | 26 (70) | 112 (66) | 118 (89) | 1,861 (69) |
| Mean BMI, kg/m^2^ | 26.59 | 26.99 | 26.75 | 26.15 | 26.13 | 27.94 | 26.47 | 26.72 | 26.00 | 26.86 | 27.12 | 26.44 | 25.70 | 26.53 |
| Exacerbations requiring antibiotics and/or OCS, within prior year,^*^ total n  n (%): 0  1  2  >2 | 155  110 (71)  22 (14)  12 (8)  11 (7) | 236  50 (21)  126 (53)  42 (18)  18 (8) | 312  92 (29)  155 (50)  38 (12)  27 (9) | 244  41 (17)  144 (59)  44 (18)  15 (6) | 299  82 (27)  133 (44)  61 (20)  23 (8) | 96  75 (78)  17 (18)  3 (3)  1 (1) | 47  32 (68)  12 (26)  2 (4)  1 (2) | 137  83 (61)  28 (20)  15 (11)  11 (8) | 171  65 (38)  44 (26)  33 (19)  29 (17) | 130  86 (66)  33 (25)  7 (5)  4 (3) | NM  NM  NM  NM  NM | 28  14 (50)  3 (11)  6 (21)  5 (18) | 133  41 (31)  43 (32)  32 (24)  17 (13) | 1,988  771 (39)  760 (38)  295 (15)  162 (8) |
| Exacerbations requiring hospitalization, within prior year,^*^ total n  n (%): 0  1  2  >2 | 156  144 (92)  11 (7)  1 (<1)  0 | 236  192 (81)  30 (13)  11 (5)  3 (1) | 312  271 (87)  33 (11)  7 (2)  1 (<1) | 244  207 (85)  31 (13)  4 (2)  2 (<1) | 299  257 (86)  32 (11)  6 (2)  4 (1) | 96  89 (93)  7 (7)  0  0 | 47  44 (94)  3 (6)  0  0 | 137  123 (90)  13 (9)  1 (<1)  0 | 171  128 (75)  34 (20)  8 (5)  1 (<1) | 130  121 (93)  7 (5)  1 (<1)  1 (<1) | NM  NM  NM  NM  NM | 28  23 (82)  5 (18)  0  0 | 133  97 (73)  28 (21)  5 (4)  3 (2) | 1,989  1,696 (85)  234 (12)  44 (2)  15 (< 1) |
| Blood neutrophil count: ≥median–<upper quartile | | | | | | | | | | | | | | |
| n | 300 | 250 | 286 | 254 | 312 | 98 | 48 | 190 | 318 | 277 | 41 | 160 | 180 | 2714 |
| Mean age, years [SD] | 63.4  [8.37] | 63.5  [9.29] | 62.0  [8.95] | 63.6  [8.82] | 63.2  [9.10] | 61.2  [8.69] | 62.0  [9.39] | 64.2  [10.19] | 64.0  [8.55] | 63.9  [8.76] | 63.8  [9.63] | 63.8  [8.53] | 65.2  [8.13] | 63.5  [8.91] |
| Gender, male, n (%) | 222 (74) | 144 (58) | 172 (60) | 162 (64) | 193 (62) | 71 (72) | 34 (71) | 139 (73) | 239 (75) | 200 (72) | 28 (68) | 121 (76) | 146 (81) | 1,871 (69) |
| Mean BMI, kg/m^2^ | 26.13 | 26.69 | 27.10 | 26.99 | 27.18 | 26.10 | 27.23 | 26.98 | 26.27 | 27.13 | 25.06 | 25.78 | 25.77 | 26.62 |
| Exacerbations requiring antibiotics and/or OCS, within prior year,^*^ total n  n (%): 0  1  2  >2 | 128  93 (73)  18 (14)  11 (9)  6 (5) | 250  51 (20)  130 (52)  45 (18)  24 (10) | 286  88 (31)  135 (47)  40 (14)  23 (8) | 254  64 (25)  134 (53)  41 (16)  15 (6) | 312  87 (28)  146 (47)  50 (16)  29 (9) | 98  73 (74)  19 (19)  6 (6)  0 | 48  33 (69)  14 (29)  1 (2)  0 | 151  99 (66)  25 (17)  17 (11)  10 (7) | 197  77 (39)  56 (28)  39 (20)  25 (13) | 119  66 (55)  25 (21)  19 (16)  9 (8) | NM  NM  NM  NM  NM | 31  11 (35)  6 (19)  8 (26)  6 (19) | 180  69 (38)  53 (29)  36 (20)  22 (12) | 2,054  811 (39)  761 (37)  313 (15)  169 (8) |
| Exacerbations requiring hospitalization, within prior year,^*^ total n  n (%): 0  1  2  >2 | 128  117 (91)  9 (7)  2 (2)  0 | 250  214 (86)  28 (11)  5 (2)  3 (1) | 286  235 (82)  49 (17)  1 (<1)  1 (<1) | 254  211 (83)  40 (16)  2 (<1)  1 (<1) | 312  266 (85)  37 (12)  6 (2)  3 (<1) | 98  91 (93)  5 (5)  2 (2)  0 | 48  43 (90)  4 (8)  1 (2)  0 | 151  135 (89)  14 (9)  0  2 (1) | 197  163 (83)  30 (15)  3 (2)  1 (<1) | 119  109 (92)  8 (7)  2 (2)  0 | NM  NM  NM  NM  NM | 31  27 (87)  3 (10)  0  1 (3) | 180  140 (78)  32 (18)  7 (4)  1 (<1) | 2,054  1,751 (85)  259 (13)  31 (2)  13 (<1) |
| Blood neutrophil count: ≥upper quartile | | | | | | | | | | | | | | |
| n | 279 | 250 | 296 | 259 | 275 | 103 | 47 | 150 | 354 | 295 | 51 | 144 | 219 | 2,722 |
| Mean age, years [SD] | 62.2  [8.53] | 63.7  [9.08] | 63.1  [9.24] | 62.0  [8.78] | 61.9  [9.35] | 60.5  [8.78] | 61.5  [9.38] | 62.5  [9.83] | 63.2  [8.73] | 63.0  [9.14] | 60.7  [9.97] | 63.6  [9.13] | 64.7  [8.07] | 62.8  [9.02] |
| Gender, male, n (%) | 201 (72) | 148 (59) | 178 (60) | 165 (64) | 179 (65) | 72 (70) | 36 (77) | 113 (75) | 282 (80) | 206 (70) | 32 (63) | 102 (71) | 188 (86) | 1,902 (70) |
| Mean BMI, kg/m^2^ | 27.00 | 27.15 | 27.29 | 27.15 | 26.98 | 26.25 | 27.22 | 27.69 | 25.93 | 26.34 | 27.05 | 26.07 | 24.99 | 26.65 |
| Exacerbations requiring antibiotics and/or OCS, within prior year,^*^ total n  n (%): 0  1  2  >2 | 117  81 (69)  22 (19)  11 (9)  3 (3) | 250  49 (20)  131 (52)  53 (21)  17 (7) | 296  92 (31)  143 (48)  43 (15)  18 (6) | 259  67 (26)  131 (51)  37 (14)  24 (9) | 275  81 (29)  126 (46)  45 (16)  23 (8) | 103  81 (79)  21 (20)  0  1 (<1) | 47  32 (68)  11 (23)  2 (4)  2 (4) | 115  72 (63)  29 (25)  9 (8)  5 (4) | 242  86 (36)  65 (27)  38 (16)  53 (22) | 136  85 (63)  30 (22)  13 (10)  8 (6) | NM  NM  NM  NM  NM | 25  7 (28)  7 (28)  6 (24)  5 (20) | 219  80 (37)  63 (29)  44 (20)  32 (15) | 2,084  813 (39)  779 (37)  301 (14)  191 (9) |
| Exacerbations requiring hospitalization, within prior year,^*^ total n  n (%): 0  1  2  >2 | 117  104 (89)  12 (10)  0  1 (<1) | 250  202 (81)  43 (17)  5 (2)  0 | 296  247 (83)  42 (14)  4 (1)  3 (1) | 259  197 (76)  59 (23)  2 (<1)  1 (<1) | 275  232 (84)  38 (14)  4 (1)  1 (<1) | 103  91 (88)  11 (11)  1 (<1)  0 | 47  39 (83)  7 (15)  1 (2)  0 | 115  102 (89)  12 (10)  1 (<1)  0 | 242  176 (73)  45 (19)  13 (5)  8 (3) | 136  116 (85)  19 (14)  0  1 (<1) | NM  NM  NM  NM  NM | 26  15 (58)  8 (31)  1 (4)  2 (8) | 219  150 (68)  49 (22)  15 (7)  5 (2) | 2,085  1,671 (80)  345 (17)  47 (2)  22 (1) |

**Abbreviations**: BMI, body mass index; FF, fluticasone furoate; NM, not measured; OCS, oral corticosteroids; SAL, salmeterol; SD, standard deviation; TIO, tiotropium; VI, vilanterol.

**Supplementary Table S3** Summary of median neutrophil counts (/mm^3^) by ICS use and study

| **Study ID** | **ICS (n=6735)** | **No ICS (n=4396)** | **Total (n=11131)** |
| --- | --- | --- | --- |
| HZC102871 | 4580.000 | 4470.000 | 4560.000 |
| HZC102970 | 4430.000 | 4430.000 | 4430.000 |
| HZC112206 | 4380.000 | 4385.000 | 4380.000 |
| HZC112207 | 4565.000 | 4600.000 | 4580.000 |
| SCO100470 | 4550.000 | 4480.000 | 4520.000 |
| SCO30002 | 4225.000 | 4029.800 | 4160.700 |
| SCO40036 | 4955.000 | 4940.000 | 4950.000 |
| SFCA3006 | 4204.190 | 4215.510 | 4208.385 |
| SFCA3007 | 4339.780 | 4544.735 | 4466.725 |
| SFCB3024 | 4420.000 | 4375.000 | 4390.000 |

**Abbreviations**: ICS, inhaled corticosteroids; ID, identifier

**Supplementary Figure S1** Boxplot of baseline neutrophils (mm^3^) by smoking status at screening


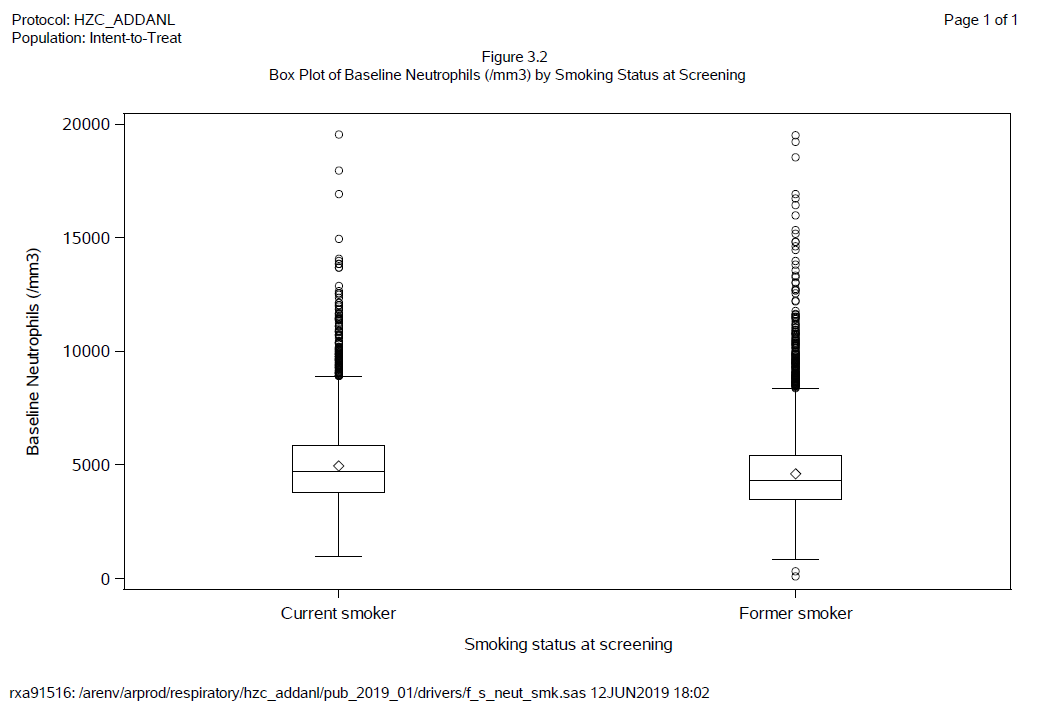

Supplement: Supplementary file 1 — Table S1. Preferred terms for pneumonia adverse events. Table S2. Patient characteristics at screening by blood neutrophil count, and treatment subgroups. Table S3. Summary of median neutrophil counts (/mm3) by ICS use and study. Figure S1. Boxplot of baseline neutrophils (mm3) by smoking status at screening. (DOCX 89 kb) [file 12931_2019_1157_MOESM1_ESM.docx]
